# Identification of mouse soleus muscle proteins altered in response to changes in gravity loading

**DOI:** 10.1038/s41598-023-42875-8

**Published:** 2023-09-22

**Authors:** Yoko Ino, Takashi Ohira, Ken Kumagai, Yusuke Nakai, Tomoko Akiyama, Kayano Moriyama, Yuriko Takeda, Tomoyuki Saito, Akihide Ryo, Yutaka Inaba, Hisashi Hirano, Yayoi Kimura

**Affiliations:** 1 Advanced Medical Research Center, Yokohama City University, Fukuura 3-9, Kanazawa-Ku, Yokohama, 236-0004 Japan; 2 Department of Physiology and Regenerative Medicine, Kindai University Faculty of Medicine, Ohno-Higashi 377-2, Osaka-Sayama, Osaka Japan; 3 Department of Orthopaedic Surgery, Yokohama City University School of Medicine, Yokohama, Japan; 4 Department of Biostatistics, Yokohama City University School of Medicine, Yokohama, Japan; 5 Yokohama Brain and Spine Center, Yokohama, Japan

**Keywords:** Proteomics, Biomarkers

## Abstract

Gravity-dependent physical processes strongly affect the ability of elderly people to maintain musculoskeletal health by reducing muscle atrophy and increasing bone mineral density, thereby increasing quality of life. A need therefore exists to identify molecules in the musculoskeletal system that are responsive to gravitational loading and to establish an objective indicator for the maintenance of healthy musculoskeletal systems. Here, we performed an integrated assessment of the results of soleus muscle proteomic analyses in three model mouse experiments under different gravity environments (hypergravity, hindlimb unloading, and spaceflight). Myl6b, Gpd1, Fbp2, Pvalb, and Actn3 were shown to be gravity-responsive muscle proteins, and alterations in the levels of these proteins indicated changes in muscle fiber type to slow-twitch type due to gravity loading. In addition, immunoblotting and enzyme-linked immunosorbent assays revealed that Pvalb levels in the sera of hindlimb-unloaded mice and osteoporosis patients were higher than in control subjects, suggesting that Pvalb levels might be useful to objectively evaluate soleus muscle atrophy and bone loss.

## Introduction

Gravity-dependent physical processes are important primary mechano-stimuli for increasing muscle mass and bone mineral density (BMD) and maintaining healthy musculoskeletal function^1^. Mice exposed to microgravity (μ-*g*) for prolonged periods during spaceflight exhibit marked changes in their musculoskeletal systems, resulting in physical and physiological deconditioning in response to physical inactivation. Gravity-dependent mechanical impacts thus play a critical role in preventing sarcopenia caused by muscle attenuation. This in turn reduces the risk of osteoporosis, a systemic condition characterized by compromised bone strength that adversely affects well-being and quality of life^2^. However, maintaining gravity-dependent physical processes is difficult during long-term spaceflight missions and in elderly people^3^. An important need exists to identify molecules in the musculoskeletal system that are responsive to gravitational unloading and to establish an objective indicator of the maintenance of healthy musculoskeletal systems.

The Multiple Artificial-gravity Research System (MARS) developed by the Japan Aerospace Exploration Agency (JAXA) allows for mice to remain in a centrifugal artificial 1 − *g* (A1 − *g*) environment aboard the International Space Station (ISS)^4^. A1-*g* exposure using centrifugation is an effective countermeasure against physical inactivation during spaceflight. Indeed, it was reported that mice bred in artificial gravity centrifuges aboard the ISS did not exhibit the bone loss or muscle atrophy induced by μ-*g* exposure during spaceflight^5^. Through comparative analysis with ground control groups, experiments in which mice remained under a centrifugal A1 − *g* environment aboard the ISS further clarified the specific response of skeletal muscle to μ − *g* exposure during spaceflight while excluding the effects of space radiation, high concentrations of carbon dioxide, and gravitational reloading upon returning to a gravitational field. Transcriptomic analysis of skeletal muscles from spaceflight mice suggested that A1-*g* exposure can suppress changes in some atrophy-related genes at the molecular level^6^. Our previous quantitative proteomic analysis using a mass spectrometer showed that compared with a μ − *g* environment, the A1 − *g* environment aboard the ISS might alter the expression profiles in the soleus muscle proteome of mice^7^. It is necessary in these studies to consider whether gene and protein expressions in the soleus muscles of mice are affected during gravitational reloading after return to Earth, even when comparing mice reared under the μ − *g* and A1 − *g* environments aboard the ISS^6,7^. The specific molecular mechanisms by which changes in gravity affect musculoskeletal systems remain unknown.

Conventional ground models in which gravity loading is decreased by hindlimb unloading (HU) and increased by centrifugation (hypergravity) have also been used to elucidate key processes in the molecular mechanism of gravity responses in the muscles and bones of mice and rats^8–13^. Prolonged muscle inactivity during HU in mice results in significant muscle atrophy and bone loss, whereas muscle mass and bone mass are increased following exposure to hypergravity. However, specific gravity-responsive molecules that might elucidate the adaptation of the musculoskeletal system to gravity loading have not been well defined to date because of the diversity of stressors that influence muscle phenotypes. HU models, for example, involve fluid shifts and behavioral restrictions^14^, whereas hypergravity models result in motion sickness^15^ and changes in the humoral immune system^16^. Thus, collectively evaluating the quantitative proteome analysis results of various experiments involving gravity loading changes might help identify proteins altered by gravity.

In this study, we attempted to identify soleus muscle proteins affected by gravity changes by performing an integrated analysis of the results of gravity change experiments under three different conditions: a μ − *g* environment aboard the ISS during long-term spaceflight, hypergravity resulting from centrifugation, and decreased gravity loading caused by HU. We narrowed down the list of proteins whose expression levels were altered in response to changes in gravity loading, and focused on parvalbumin (Pvalb), a secreted protein with calcium-ion binding structures. Further experiments revealed that Pvalb levels were increased in the sera of HU mice and patients with osteoporosis compared with control subjects, suggesting that Pvalb might be a new candidate as an objective indicator for the evaluation of soleus muscle atrophy and bone loss.

## Results

### Discovery of gravity-responsive proteins in soleus muscles of mice

**Figure 1:**
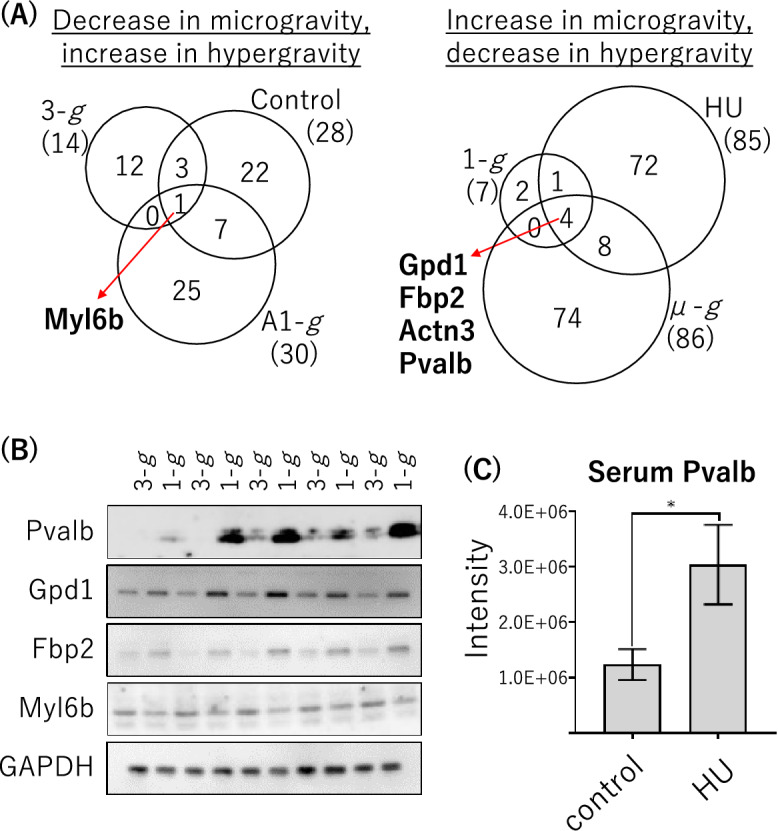
Identification of mice soleus muscle proteins in response to changes in gravity loading. (**A**) Proteins with significantly decreased or increased levels (< 0.01, fold change ≤ 0.5 or ≥ 2), as compared to control, in three gravity change experiments in model mice. (**B**) The level of each soleus muscle protein from the hypergravity and control mice. The immunoblotting analysis procedure for protein detection is described in the Materials and Methods. Note that the membrane is cropped to remove irrelevant areas, and uncropped images are shown in Supplemental Fig. S2. (**C**) Serum Pvalb levels from HU and control mice as detected by immunoblot analysis (Supplemental Fig. S4). The protein levels were determined from the band intensity using Multi Gauge software (*p < 0.05).

To identify unique soleus muscle proteins that respond to gravity changes, we evaluated the results of three experiments (involving hypergravity, HU, or spaceflight), and detected protein alterations with quantitative proteome analysis using mass spectrometry (MS). Body weights and muscle wet weights of mice from each experiment are shown in Supplemental Table S1. Muscle weight increased under elevated gravity and decreased under reduced gravity, with the soleus muscle weight being particularly responsive to gravitational change. Proteins from the soleus muscles of mice were extracted into soluble and insoluble fractions, and each was subsequently subjected to quantitative MS analysis. In the present study, quantitative analysis of the hypergravity and spaceflight experiments was performed using MS data from mouse soleus muscles reported in our previous studies^7,13^. In statistical analysis using Progenesis QI for proteomics, the threshold criterion for both increased and decreased proteins was set at *p* < 0.01, which was considered statistically significant, and the fold change was set at ≤ 0.5 or ≥ 2, compared to each control in quantitative analyses. In the hypergravity experiment, the levels of 21 soleus muscle proteins were substantially altered in mice in a 3 − *g* environment compared with those in a 1 − *g* environment (Supplemental Table S2). The levels of 116 soleus muscle proteins differed substantially between spaceflight mice exposed to a μ − *g* environment and those exposed to the centrifugal A1-*g* environment aboard the ISS (Supplemental Table S3). An additional analysis revealed that the levels of 113 soleus muscle proteins differed between HU and control mice (Supplemental Table S4). More soleus muscle proteins were increased or decreased in mice in reduced gravity environments (HU and spaceflight) than in those in a hypergravity environment. Gene ontology analysis was performed to characterize the biological processes associated with proteins whose expression levels were altered in each experiment. Most of the biological processes were specific to each experiment (Supplemental Table S5), suggesting that a variety of factors significantly affected protein expression levels in each of the three conditions. On the other hand, some common biological processes were identified, such as muscle contraction, muscle filament sliding, and gluconeogenesis. Therefore, an analysis integrating the results of these experiments was expected to determine which proteins associated with muscle contraction function were common to all three experiments. Finally, an evaluation of the quantitative results of the three types of experiments identified five potential gravity-responsive proteins (Fig. 1A, Table 1, and Supplemental Fig. S1). The only protein that increased in response to gravity loading was myosin light chain 6B (Myl6b), which is the myosin light chain isoform in slow-twitch muscle tissue^17,18^. The proteins that decreased in response to gravity loading changes were proteins abundant in fast-twitch muscle tissue: glycerol-3-phosphate dehydrogenase [NAD( +)] (Gpd1), fructose-1,6-bisphosphatase isozyme 2 (Fbp2), parvalbumin alpha (Pvalb), and alpha-actinin-3 (Actn3). In addition, Myl6b and Actn3 were detected in both the soluble and insoluble fractions of soleus muscle (Table 1). In the fast-twitch extensor digitorum longus (EDL) of spaceflight and hypergravity mice, no significant gravity-induced increases or decreases (*p* < 0.01, fold change ≤ 0.5 or ≥ 2) in the levels of these five proteins were observed^7,13^ (Supplemental Table S6). Further immunoblotting analysis showed that the levels of Pvalb, Gpd1, Fbp2, and Myl6b in the mouse soleus muscle were altered by changes in gravity loading in hypergravity experiments (Fig. 1B and Supplemental Fig. S2). These results were consistent with those of the MS analysis, suggesting that these protein levels, which were detected based on an integrated evaluation of the three gravity change experiments, are responsive to gravity loading changes^19–25^. Previous studies on these proteins have shown that Gpd1^26^ and Fbp2^27^ are involved in glucose metabolism, Actn3^28^ is a muscle structural protein, and Pvalb^29,30^ is a Ca^2+^-binding protein. In the present study, we specifically focused on the secreted protein Pvalb, which might be involved in the bone loss associated with muscle atrophy, and performed the following studies.

**Table 1:** Five proteins exhibiting gravity-associated changes in levels in all three experiments with different gravity loading conditions (*p* < 0.01, fold change ≤ 0.5 or ≥ 2).

Accession	Gene name	Description	Unique peptides	Confidence score	*p*-value	Fold change		Fractions*
Q8CI43	Myl6b	Myosin light chain 6B	15	1299.7	5.872E − 05	3-*g*/1-*g*	2.06	Sol/Ins
			10	728.9	1.718E − 04	Control/HU	2.61	Sol/Ins
			13	1094.9	2.114E − 05	A1-*g*/μ-*g*	2.68	Sol/Ins
P13707	Gpd1	Glycerol-3-phosphate dehydrogenase [NAD( +)], cytoplasmic	7	498.1	6.760E − 05	3-*g*/1-*g*	0.44	Sol
			10	784.6	8.360E − 05	Control/HU	0.38	Sol
			22	1733.3	1.248E − 09	A1-*g*/μ-*g*	0.32	Sol
P70695	Fbp2	Fructose-1,6-bisphosphatase isozyme 2	3	255.0	4.579E − 04	3-*g*/1-*g*	0.43	Sol
			3	270.2	3.819E − 03	Control/HU	0.46	Sol
			3	215.2	4.077E − 05	A1-*g*/μ-*g*	0.30	Sol
O88990	Actn3	Alpha-actinin-3	27	2845.4	2.528E − 03	3-*g*/1-*g*	0.24	Ins
			37	3579.8	1.480E − 03	Control/HU	0.28	Sol/Ins
			45	5232.1	1.493E − 05	A1-*g*/μ-*g*	0.41	Sol/Ins
P32848	Pvalb	Parvalbumin alpha	7	368.9	3.440E − 03	3-*g*/1-*g*	0.32	Sol
			6	374.5	6.708E − 03	Control/HU	0.41	Sol
			5	231.3	1.397E − 04	A1-*g*/μ-*g*	0.34	Sol

*Identified in soluble (Sol) or insoluble (Ins) fractions.

### Serum Pvalb levels in response to gravity loading changes

Pvalb is mainly expressed in endocrine tissues rather than muscle tissues, as shown in the Human Protein Atlas (https://www.proteinatlas.org/). Indeed, Pvalb expressed in C2C12 muscle cells as a result of plasmid transfection was detected in the culture medium as well as in the cell extract (Supplemental Fig. S3). We next examined Pvalb levels in the serum of HU mice, a model of soleus muscle atrophy caused by gravity unloading of the hindlimbs. As in muscle tissue, Pvalb levels in the serum of HU mice were increased compared to those of control mice (Fig. 1C and Supplemental Fig. S4). A previous immunohistochemical study also identified Pvalb in the extracellular matrix of calcified cartilage in rats^31^. In addition, Pvalb, a calcium ion–binding protein with two EF-hand domains at the C-terminus^32–36^, is known to be involved in the regulation of free calcium ion buffering intracellularly in both cartilage and bone cells. Gene-deficient mice have shown a positive calcium ion balance and increased BMD^37^. Thus, Pvalb, which is a protein that is decreased in response to gravity loading, might be involved in regulating the gravity-dependent physical processes that are important for maintaining bone health, as reflected by BMD^38^. To examine trabecular and cortical femoral bone morphology in HU mice, which exhibited increased serum Pvalb levels, we assessed the bone microstructure using microcomputed tomography^39^. For the trabecular bone analysis, the distal metaphyseal femoral regions were scanned, and the following parameters were assessed: bone volume fraction (BV/TV), trabecular thickness (Tb.Th), trabecular number (Tb.N), and trabecular separation (Tb.Sp). For the cortical bone analysis, the mid-shaft femoral regions were scanned, and the following parameters were assessed: cortical thickness (Ct.Th) and cortical area (Ct.Ar). The results showed that HU was associated with decreased BV/TV, Tb.Th, Tb.N, Ct.Th, and Ct.Ar, and with increased Tb.Sp. These results indicate that bone loss and fragility were induced in HU mice (Supplemental Fig. S5). Therefore, we subsequently investigated the relationship between increased serum Pvalb levels and osteoporosis, which is one of the bone loss diseases caused by gravity changes^40,41^.

**Figure 2:**
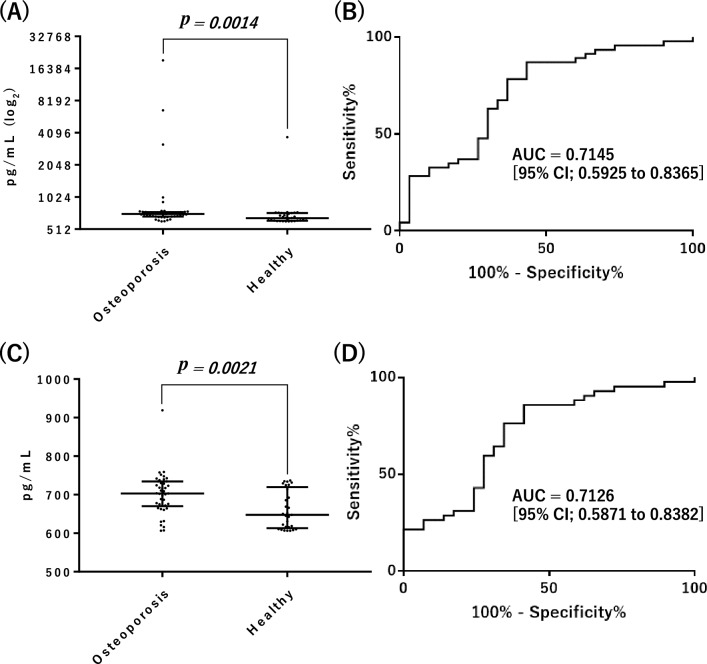
Serum levels and ROC curves of Pvalb, as determined by ELISA. (**A**) Left, in 46 osteoporosis patients, the median serum level [interquartile range, IQR] of Pvalb was 709.8 [672.7–739.3] pg/mL. Right, in 30 healthy subjects, the median serum level [IQR] of Pvalb was 649.0 [616.0–722.3] pg/mL. Horizontal bars represent the median and IQR. (**B**) ROC curve for discriminating between patient groups. “AUC” indicates the area under the ROC curve [95% CI]. (**C**) Pvalb serum levels derived using samples excluding those from four patients and one healthy subject with particularly high levels of Pvalb (> 1 ng/mL). Left, in 42 osteoporosis patients, the median serum level [IQR] of Pvalb was 703.2 [671.7–733.2] pg/mL. Right, in 29 healthy subjects, the median serum level [IQR] of Pvalb was 647.6 [615.8–714.4] pg/mL. Horizontal bars represent the median and IQR. (**D**) ROC curve for discriminating patient groups, excluding results of samples from four patients and one healthy subject with particularly high levels of Pvalb (> 1 ng/mL). “AUC” indicates the area under the ROC curve [95% CI].

We used an enzyme-linked immunosorbent assay (ELISA) to compare serum levels of Pvalb in 46 patients with osteoporosis (defined by a T-score ≤ − 2.5 according to the definition of the World Health Organization^42^) and 30 healthy subjects. The clinical information of the patients enrolled in this study is presented in Supplemental Table S7. Differences were considered to be statistically significant for *p* < 0.01 (Mann–Whitney U-test). The serum level of Pvalb was significantly different between osteoporosis patients and healthy subjects (*p* = 0.0014, Fig. 2A). We further used receiver operating characteristic (ROC) curve analysis to assess the ability of Pvalb to discriminate between osteoporosis patients and healthy subjects. In the present study set, the area under the ROC curve (AUC) [95% confidence intervals (CIs)] of this model using Pvalb levels was 0.7145 [0.5925–0.8365] (Fig. 2B). Even when comparing 42 patients and 29 healthy subjects, after excluding four patients and one healthy subject with particularly high levels of Pvalb (> 1 ng/mL), the Pvalb level was higher in the patients (*p* = 0.0021, Fig. 2C) and the AUC [95% CI] of this model was 0.7126 [0.5871–0.8382] (Fig. 2D). These results suggest that the serum level of Pvalb, in addition to BMD, might serve as an objective indicator for the evaluation of soleus muscle atrophy and bone loss. However, we identified no clear correlation between T-score and Pvalb level in patients in the present study set (Spearman correlation coefficient, *r* =  − 0.095).

## Discussion

Gravity-dependent physical processes are critical mechano-stimulants that can help maintain healthy musculoskeletal function and thereby prevent sarcopenia, defined as muscle attenuation, and osteoporosis, which compromises bone strength. To identify soleus muscle proteins that responded to gravity loading changes, we performed an integrated assessment of quantitative proteomic analyses of mouse experiments involving three types of gravity changes: A1 − *g* and μ − *g* environments aboard the ISS during long-term spaceflight, hyper gravity caused by centrifugation, and decreased gravity loading resulting from HU. Using this approach, we discovered five gravity-responsive proteins, namely Myl6b, Gpd1, Fbp2, Pvalb, and Actn3. Altered expression levels of these proteins have also been reported in mouse models of denervation, aging, and dystrophy^43–46^. Bone and muscle are attached to each other, and their relationship has been thought to be solely mechanical; however, researchers have recently proposed that they are both secretory tissues that regulate each other’s metabolism^47–49^. Proteins secreted by muscle, collectively referred to as myokines, appear to be important regulators of bone quantity and quality as a result of affecting osteoblast and osteoclast differentiation. Pvalb, whose expression was found to be decreased by gravity loading in this study, is known to be a protein with two calcium ion binding structures (EF hands) similar to calmodulin and troponin C^50,51^. Increased Pvalb levels induce a decrease in intracellular calcium ions^52^, and calcium ion deficiency leads to a reduction in BMD and, in turn, to osteoporosis^53^. Indeed, in the present study, Pvalb protein levels were increased in the serum of HU mice with soleus muscle atrophy and bone loss. Additionally, overexpression of Pvalb was reported to decrease the number of calcium ions taken up by mitochondria, which in turn caused muscle atrophy, and Pvalb knockout did not affect the ability of Akt activation^54^. Microgravity-induced muscle atrophy and bone loss is deeply implicated in the pathology of osteoporosis^41^ and sarcopenia^55^. In fact, this study identified higher levels of Pvalb in patients with osteoporosis (specifically, T-score ≤ − 2.5) than in healthy subjects, although no clear correlation was observed between the T-score and Pvalb serum levels. Further studies are needed to elucidate whether mechanisms of Pvalb regulation directly or indirectly influence bone metabolism. An additional future goal is to determine if Pvalb affects the regulation of soleus muscle mass.

The results of this study indicate that Pvalb might be a new candidate as an objective indicator for the evaluation of soleus muscle atrophy and bone loss, and if so, this would help promote the maintenance of healthy musculoskeletal systems. However, this study has several limitations, especially in the verification analysis, where we used a small sample size to compare the serum levels of Pvalb between osteoporosis patients and healthy subjects. In addition, the patient information did not include body mass index or skeletal muscle mass index. Further validation using serum from patients with diverse backgrounds is needed to evaluate the clinical usefulness of Pvalb as an objective indicator for the evaluation of soleus muscle atrophy and bone loss.

## Materials and methods

### Experimental animals for HU experiments

All experimental procedures were conducted in accordance with the “Guide for the Care and Use of Laboratory Animals” of the Japanese Physiological Society, and the “Guide for the Care and Use of Laboratory Animals” of the National Institutes of Health and ARRIVE guidelines. C57BL/6 J mice (9–10 weeks old; Chubu Kagaku Shizai, Nagoya, Japan) were used in all three gravity studies. Hindlimb unloading of mice (n = 5) was conducted in reference to a previous report^56^ with slight modifications. The mice were anesthetized with 2% isoflurane, and their tails were sterilized with povidone-iodine solution. A 2–0 sterile surgical steel wire was passed through the fifth intervertebral disc of the tail (counting from end of the body) and formed into a ring, which was lifted upward to keep the hindlimbs off the floor of the cage. Mice in the control group also underwent steel wire insertion through the tail, but the hindlimbs were not lifted off the floor. The mice in each cage accessed food and water ad libitum throughout the 42-day study period. This study was approved by the Committee on Animal Care and Use of Yokohama City University (accreditation no.: F-A-16-066). The hypergravity and spaceflight mouse experiments were conducted for 28 and 30 days, respectively, and were described elsewhere^7,13^. Female and male mice were used in the HU experiment and the hypergravity/spaceflight experiments, respectively.

### Proteomic analysis using DDA-MS analysis

The sample preparation procedures for MS analysis, data-dependent acquisition mode mass spectrometry (DDA-MS) analysis, and data analysis were conducted in accordance with previous studies^7,13^. Nonlabeled quantitative and statistical analyses were performed with the Progenesis QI for proteomics software (version 4.2; Nonlinear Dynamics, Newcastle, UK) using MS data of extracted proteins in soluble and insoluble fractions from soleus muscles to generate one output file. All settings were set to default.

### Immunoblotting analysis

Proteins extracted from the soleus muscle in the hypergravity mouse experiment were separated by SDS-PAGE followed by electroblotting onto polyvinylidene fluoride (PVDF) membranes. Sera in the HU mouse experiment were used for immunoblotting analysis after the removal of seven mouse proteins (albumin, IgG, alpha1-antitrypsin, IgM, transferrin, haptoglobin and fibrinogen) using a Seppro Mouse Spin Column (Sigma-Aldrich, St. Louis, MO). Serum proteins were separated by SDS-PAGE followed by Coomassie brilliant blue (CBB) staining or electroblotting onto PVDF. After blocking with 5% nonfat dry milk in Tris-buffered saline containing Tween 20, the membrane was incubated with anti-Pvalb antibody (1:2000, Proteintech, Rosemont, IL, USA), Myl6b (1:1000, Abnova, Taipei, Taiwan), Gpd1 (1:1000, Proteintech), Fbp2 (1:1000, Proteintech), and GAPDH (1:3000, Cell Signaling Technology, Danvers, MA, USA), followed by incubation with horseradish peroxidase–conjugated secondary antibodies. GAPDH was used as the endogenous control. Signals were visualized using the ECL Select Western Blotting Detection Reagent (GE Healthcare, Chicago, IL, USA) and an LAS-4000 miniature luminescent image analyzer (FUJIFILM, Tokyo, Japan). Quantitation of detected bands was performed using Multi Gauge software (version 3.11, FUJIFILM), and *p* values were calculated by the Mann–Whitney U-test using GraphPad Prism software (version 7.0.2, GraphPad Software Inc., San Diego, CA, USA).

### ELISA analysis of human serum samples

Serum samples were obtained from osteoporosis patients (lumbar vertebral T-score ≤  − 2.5) who attended Yokohama City University Hospital between September 2016 and August 2018 or from otherwise healthy volunteers (employees of Yokohama City University) from January 2014 to December 2015. This research protocol was approved by the Clinical Ethics Committee of Yokohama City University Hospital (B160401019). Written informed consent was obtained from all patients or their guardians before serum sample collection. This study was conducted in accordance with the Declaration of Helsinki. All data were anonymized before the analyses. Serum Pvalb concentrations were determined using an ELISA kit (Signalway Antibody, College Park, MA, USA). The absorbance was measured at 450 nm using an Infinite 200 microplate reader (Tecan, Mannedörf, Switzerland). The Mann–Whitney U-test and ROC curve analysis were performed using GraphPad Prism software to determine statistical significance between the osteoporosis group and healthy controls.

### Supplementary Information

Supplementary Figures.

Supplementary Tables.

## Data Availability

The datasets generated or analyzed during the current study are available in the ProteomeXchange Consortium (http://www.proteomexchange.org) via the jPOST (https://jpostdb.org) partner repository with the dataset identifier PXD041190. All data are fully available without restriction.

## References

[1] Reginster JY, Beaudart C, Buckinx F, Bruyere O (2016). Osteoporosis and sarcopenia: Two diseases or one?. Curr. Opin. Clin. Nutr. Metab. Care.

[2] Phu S, Boersma D, Duque G (2015). Exercise and sarcopenia. J. Clin. Densitom..

[3] Hargens AR, Bhattacharya R, Schneider SM (2013). Space physiology VI: Exercise, artificial gravity, and countermeasure development for prolonged space flight. Eur. J. Appl. Physiol..

[4] Shiba D (2017). Development of new experimental platform 'MARS'-multiple artificial-gravity research system-to elucidate the impacts of micro/partial gravity on mice. Sci. Rep..

[5] Furukawa S (2021). Findings from recent studies by the Japan Aerospace Exploration Agency examining musculoskeletal atrophy in space and on Earth. NPJ Microgravity.

[6] Okada R (2021). Transcriptome analysis of gravitational effects on mouse skeletal muscles under microgravity and artificial 1 g onboard environment. Sci. Rep..

[7] Ohira T (2021). Effects of microgravity exposure and fructo-oligosaccharide ingestion on the proteome of soleus and extensor digitorum longus muscles in developing mice. NPJ Microgravity.

[8] Kawao N (2016). The vestibular system is critical for the changes in muscle and bone induced by hypergravity in mice. Physiol. Rep..

[9] Kawao N, Morita H, Obata K, Tatsumi K, Kaji H (2018). Role of follistatin in muscle and bone alterations induced by gravity change in mice. J. Cell. Physiol..

[10] Laurent MR (2016). Muscle-bone interactions: From experimental models to the clinic? A critical update. Mol. Cell. Endocrinol..

[11] Mirzoev T, Tyganov S, Vilchinskaya N, Lomonosova Y, Shenkman B (2016). Key markers of mTORC1-dependent and mTORC1-independent signaling pathways regulating protein synthesis in rat soleus muscle during early stages of hindlimb unloading. Cell. Physiol. Biochem..

[12] Nakagawa T (2018). The effects of bisphosphonate on pain-related behavior and immunohistochemical analyses in hindlimb-unloaded mice. J. Orthop. Sci..

[13] Ohira T (2020). Proteomic analysis revealed different responses to hypergravity of soleus and extensor digitorum longus muscles in mice. J. Proteomics.

[14] Globus RK, Morey-Holton E (2016). Hindlimb unloading: Rodent analog for microgravity. J. Appl. Physiol..

[15] Santucci D (2000). Neurobehavioural effects of hypergravity conditions in the adult mouse. NeuroReport.

[16] Gueguinou N (2012). Stress response and humoral immune system alterations related to chronic hypergravity in mice. Psychoneuroendocrinology.

[17] Maire P (2020). Myogenesis control by SIX transcriptional complexes. Semin. Cell Dev. Biol..

[18] Pathmanathan S (2008). IQ motif selectivity in human IQGAP1: Binding of myosin essential light chain and S100B. Mol. Cell. Biochem..

[19] Adams GR, Caiozzo VJ, Baldwin KM (2003). Skeletal muscle unweighting: Spaceflight and ground-based models. J. Appl. Physiol..

[20] Fitts RH (2010). Prolonged space flight-induced alterations in the structure and function of human skeletal muscle fibres. J. Physiol..

[21] Narici MV, de Boer MD (2011). Disuse of the musculo-skeletal system in space and on earth. Eur. J. Appl. Physiol..

[22] Ohira T, Kawano F, Ohira T, Goto K, Ohira Y (2015). Responses of skeletal muscles to gravitational unloading and/or reloading. J. Physiol. Sci..

[23] Ohira T (2011). Region-specific responses of adductor longus muscle to gravitational load-dependent activity in Wistar Hannover rats. PLoS One.

[24] Qaisar R, Karim A, Elmoselhi AB (2020). Muscle unloading: A comparison between spaceflight and ground-based models. Acta Physiol. (Oxf.).

[25] Trappe S (2009). Exercise in space: Human skeletal muscle after 6 months aboard the International Space Station. J. Appl. Physiol..

[26] Okumura N (2005). Proteomic analysis of slow- and fast-twitch skeletal muscles. Proteomics.

[27] Cadefau JA, Parra J, Tauler A, Cusso R (1999). Contractile activity modifies Fru-2,6-P(2) metabolism in rabbit fast twitch skeletal muscle. J. Biol. Chem..

[28] Vincent B (2007). ACTN3 (R577X) genotype is associated with fiber type distribution. Physiol. Genomics.

[29] Celio MR, Heizmann CW (1982). Calcium-binding protein parvalbumin is associated with fast contracting muscle-fibers. Nature.

[30] Schwaller B (1999). Prolonged contraction-relaxation cycle of fast-twitch muscles in parvalbumin knockout mice. Am. J. Physiol..

[31] Celio MR, Norman AW, Heizmann CW (1984). Vitamin-D-dependent calcium-binding-protein and parvalbumin occur in bones and teeth. Calcified Tissue Int..

[32] Celio MR, Heizmann CW (1982). Calcium-binding protein parvalbumin is associated with fast contracting muscle fibres. Nature.

[33] Endo T, Takazawa K, Onaya T (1985). Parvalbumin exists in rat endocrine glands. Endocrinology.

[34] Heizmann CW, Berchtold MW, Rowlerson AM (1982). Correlation of parvalbumin concentration with relaxation speed in mammalian muscles. Proc. Natl. Acad. Sci. U. S. A..

[35] Kagi U, Berchtold MW, Heizmann CW (1987). Ca2+-binding parvalbumin in rat testis. Characterization, localization, and expression during development. J. Biol. Chem..

[36] Pechere JF, Derancourt J, Haiech J (1977). The participation of parvalbumins in the activation-relaxation cycle of vertebrate fast skeletal-muscle. FEBS Lett..

[37] Belge H (2007). Renal expression of parvalbumin is critical for NaCl handling and response to diuretics. Proc. Natl. Acad. Sci. U. S. A..

[38] Shackelford LC (2004). Resistance exercise as a countermeasure to disuse-induced bone loss. J. Appl. Physiol..

[39] Bouxsein ML (2010). Guidelines for assessment of bone microstructure in rodents using micro-computed tomography. J. Bone Miner. Res..

[40] Birge SJ, Dalsky G (1989). The role of exercise in preventing osteoporosis. Public Health Rep..

[41] Herrmann M (2020). Interactions between muscle and bone-where physics meets biology. Biomolecules.

[42] Binkley N, Adler R, Bilezikian JP (2014). Osteoporosis diagnosis in men: The T-score controversy revisited. Curr. Osteoporos Rep..

[43] Lang F (2018). Single muscle fiber proteomics reveals distinct protein changes in slow and fast fibers during muscle atrophy. J. Proteome Res..

[44] Seo JY (2021). Maintenance of type 2 glycolytic myofibers with age by Mib1-Actn3 axis. Nat. Commun..

[45] Tascher G (2017). Proteome-wide adaptations of mouse skeletal muscles during a full month in space. J. Proteome Res..

[46] Jockusch H, Friedrich G, Zippel M (1990). Serum parvalbumin, an indicator of muscle disease in murine dystrophy and myotonia. Muscle Nerve.

[47] Cariati I (2021). Role of physical activity in bone-muscle crosstalk: Biological aspects and clinical implications. J. Funct. Morphol. Kinesiol..

[48] Kirk B, Feehan J, Lombardi G, Duque G (2020). Muscle, bone, and fat crosstalk: The biological role of myokines, osteokines, and adipokines. Curr. Osteoporos. Rep..

[49] Li G (2019). Muscle-bone crosstalk and potential therapies for sarco-osteoporosis. J. Cell. Biochem..

[50] Arif SH (2009). A Ca(2+)-binding protein with numerous roles and uses: Parvalbumin in molecular biology and physiology. BioEssays.

[51] Cates MS (1999). Metal-ion affinity and specificity in EF-hand proteins: Coordination geometry and domain plasticity in parvalbumin. Structure.

[52] Muller M, Felmy F, Schwaller B, Schneggenburger R (2007). Parvalbumin is a mobile presynaptic Ca2+ buffer in the calyx of Held that accelerates the decay of Ca2+ and short-term facilitation. J. Neurosci..

[53] Rodriguez-Martinez MA, Garcia-Cohen EC (2002). Role of Ca(2+) and vitamin D in the prevention and treatment of osteoporosis. Pharmacol. Ther..

[54] Butera G (2021). Parvalbumin affects skeletal muscle trophism through modulation of mitochondrial calcium uptake. Cell Rep..

[55] Kawao N, Kaji H (2015). Interactions between muscle tissues and bone metabolism. J. Cell Biochem..

[56] Ferreira JA, Crissey JM, Brown M (2011). An alternant method to the traditional NASA hindlimb unloading model in mice. J. Vis. Exp..

