# Supplementary material for: Identification of mouse soleus muscle proteins altered in response to changes in gravity loading
Source: Sci Rep. 2023 Sep 22;13:15768. doi: 10.1038/s41598-023-42875-8 (PMC10517164; doi:10.1038/s41598-023-42875-8)
Supplement: Supplementary file 1 — Supplementary Figures. [file 41598_2023_42875_MOESM1_ESM.pptx]

## Slide 1
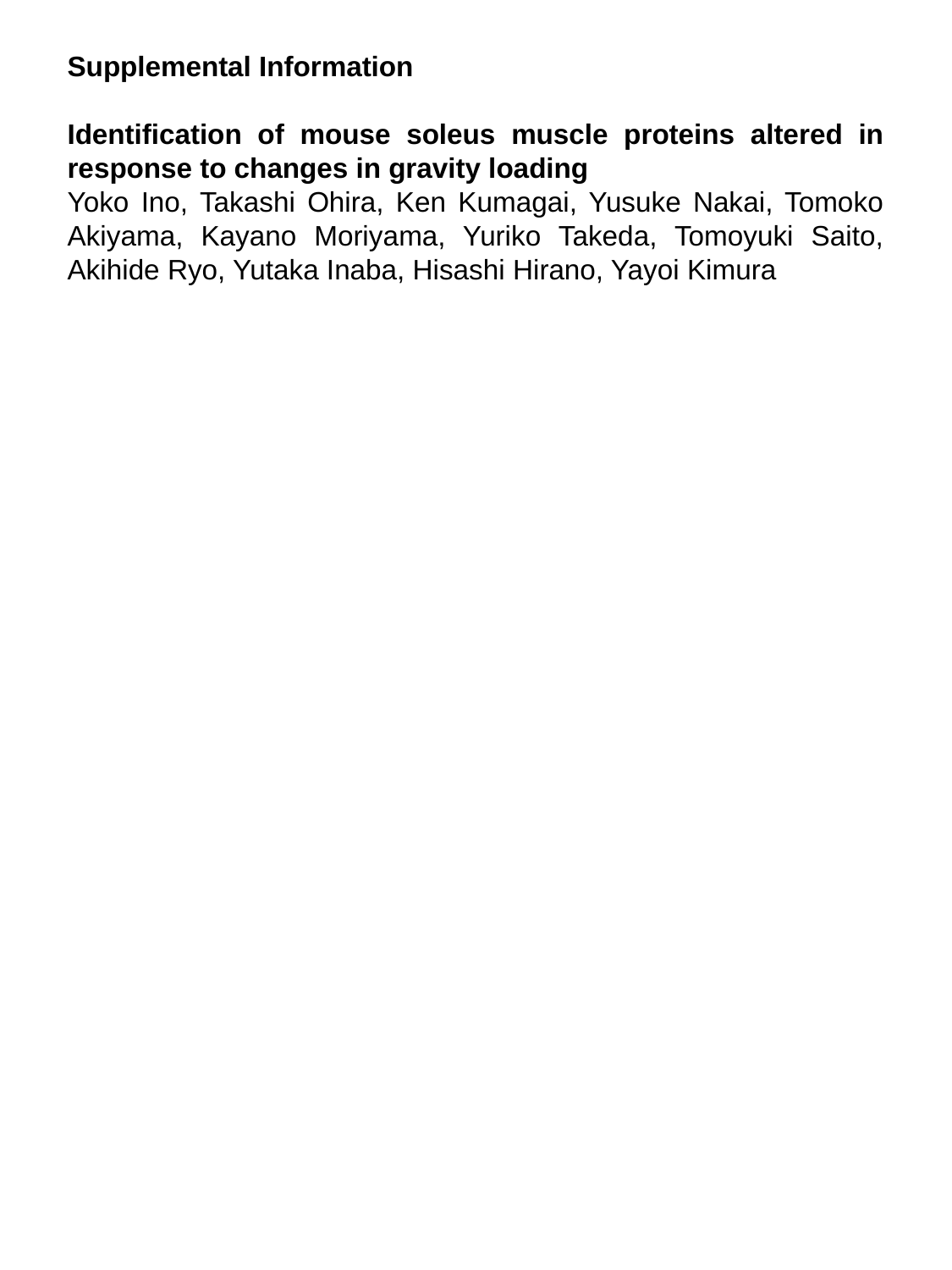

Supplemental Information
Identification of mouse soleus muscle proteins altered in response to changes in gravity loading
Yoko Ino, Takashi Ohira, Ken Kumagai, Yusuke Nakai, Tomoko Akiyama, Kayano Moriyama, Yuriko Takeda, Tomoyuki Saito, Akihide Ryo, Yutaka Inaba, Hisashi Hirano, Yayoi Kimura

## Slide 2
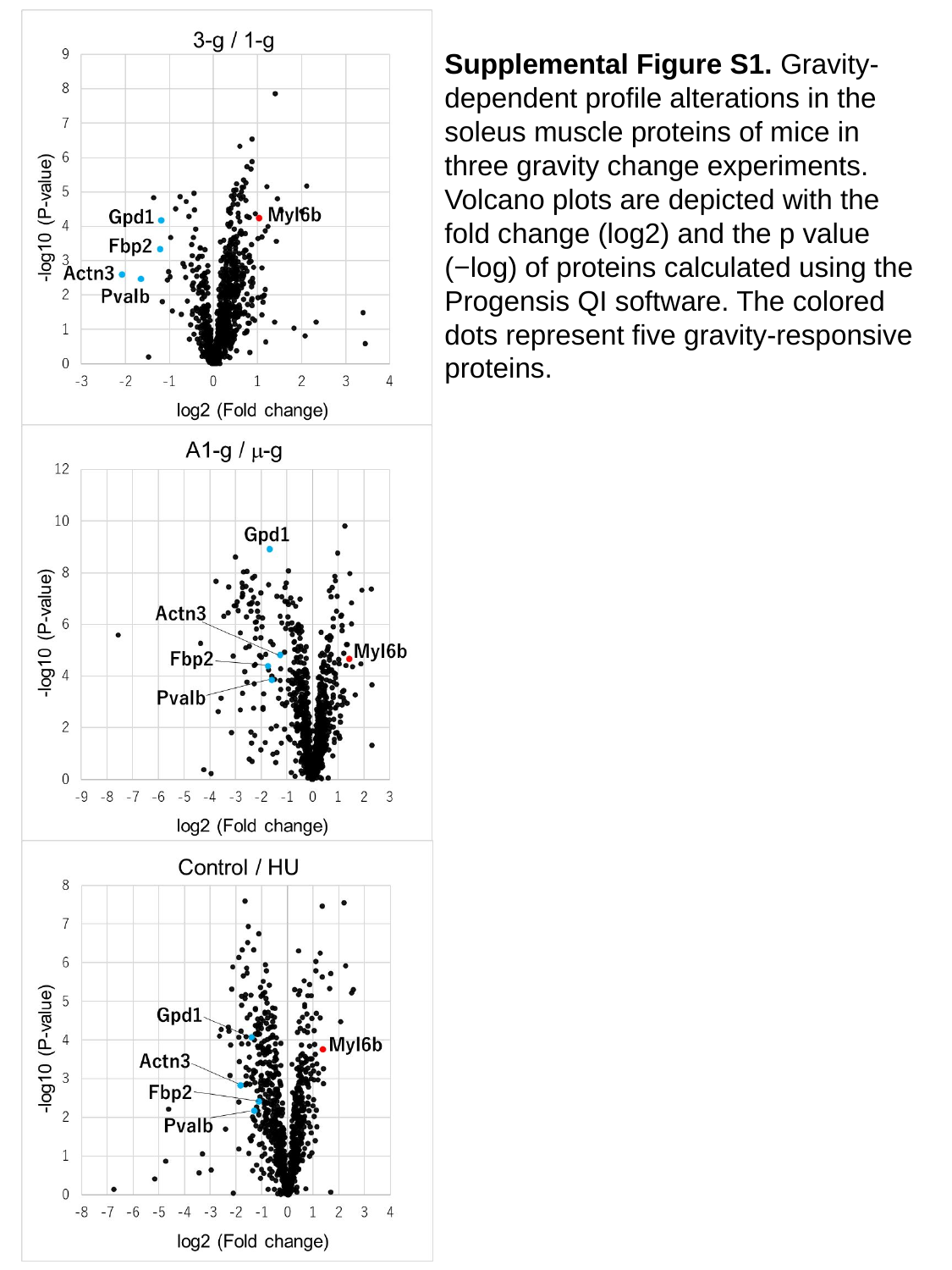

Supplemental Figure S1. Gravity-dependent profile alterations in the soleus muscle proteins of mice in three gravity change experiments. Volcano plots are depicted with the fold change (log2) and the p value (−log) of proteins calculated using the Progensis QI software. The colored dots represent five gravity-responsive proteins.

## Slide 3
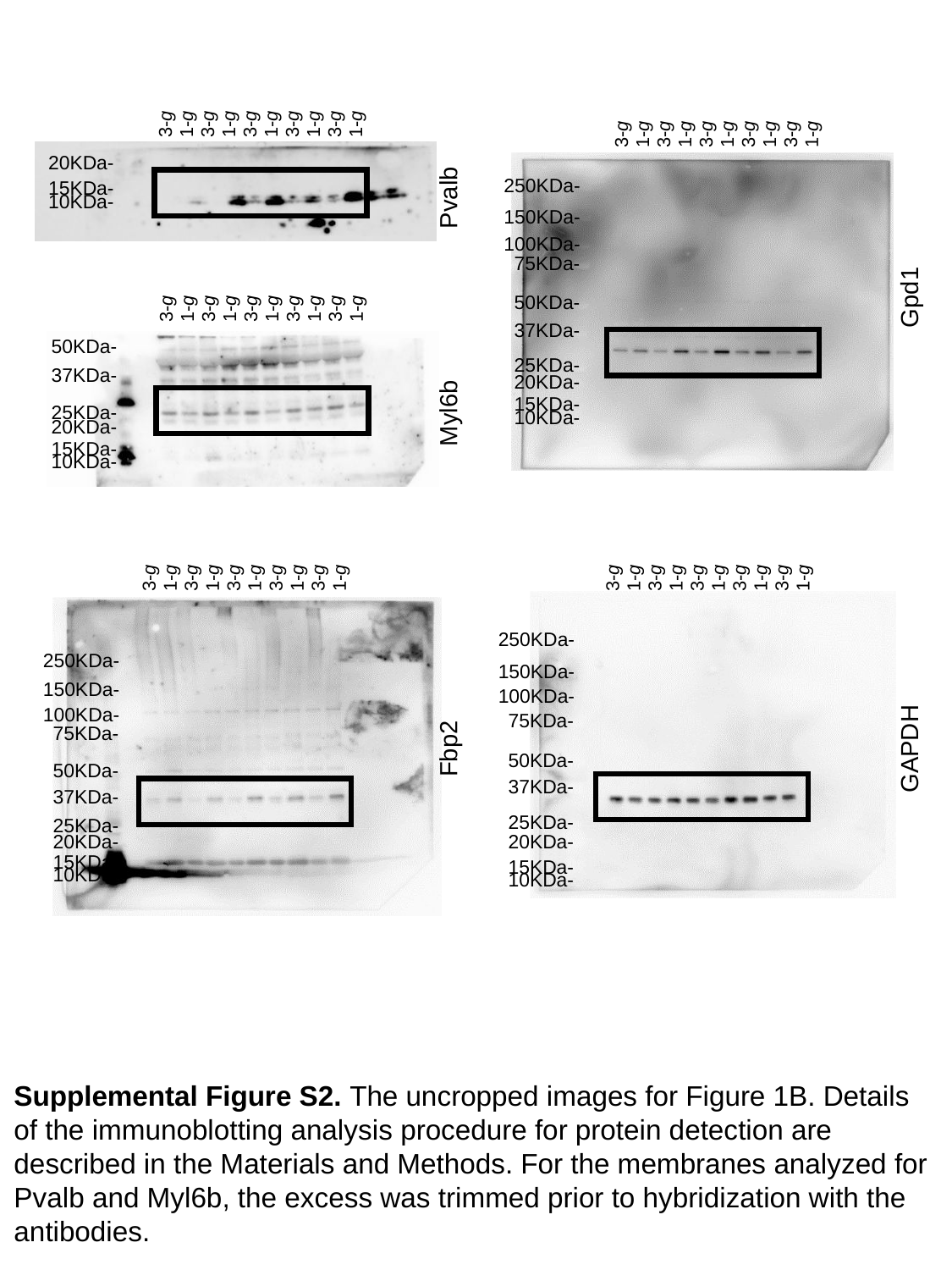

3-g
1-g
3-g
1-g
3-g
1-g
3-g
1-g
3-g
1-g
3-g
1-g
3-g
1-g
3-g
1-g
3-g
1-g
3-g
1-g
20KDa-
250KDa-
15KDa-
Pvalb
10KDa-
3-g
1-g
3-g
1-g
3-g
1-g
3-g
1-g
3-g
1-g
150KDa-
100KDa-
75KDa-
Gpd1
50KDa-
37KDa-
50KDa-
25KDa-
37KDa-
20KDa-
15KDa-
Myl6b
25KDa-
10KDa-
20KDa-
15KDa-
10KDa-
3-g
1-g
3-g
1-g
3-g
1-g
3-g
1-g
3-g
1-g
3-g
1-g
3-g
1-g
3-g
1-g
3-g
1-g
3-g
1-g
250KDa-
250KDa-
150KDa-
150KDa-
100KDa-
100KDa-
75KDa-
75KDa-
Fbp2
GAPDH
50KDa-
50KDa-
37KDa-
37KDa-
25KDa-
25KDa-
20KDa-
20KDa-
15KDa-
15KDa-
10KDa-
10KDa-
Supplemental Figure S2. The uncropped images for Figure 1B. Details of the immunoblotting analysis procedure for protein detection are described in the Materials and Methods. For the membranes analyzed for Pvalb and Myl6b, the excess was trimmed prior to hybridization with the antibodies.

## Slide 4
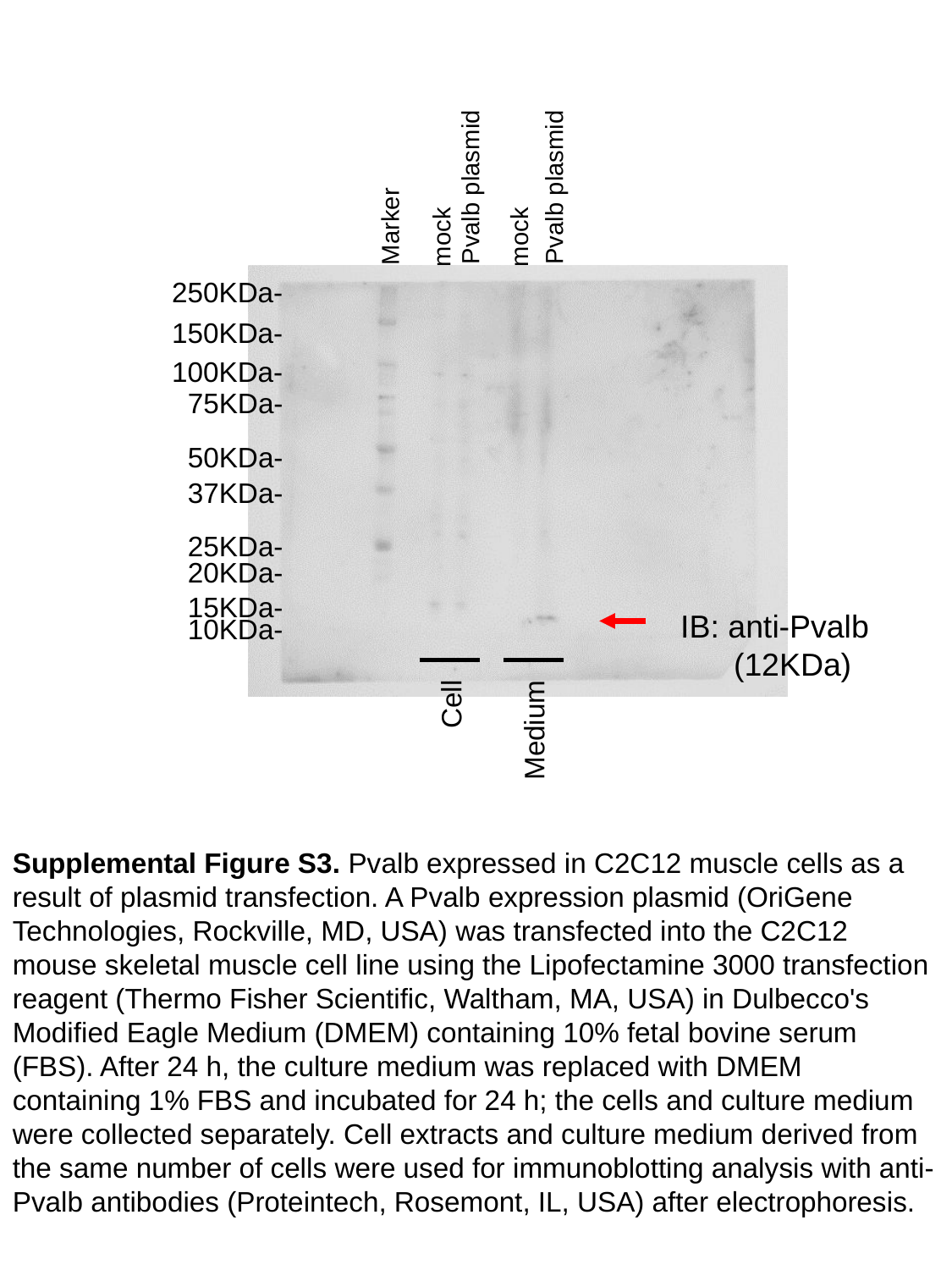

Pvalb plasmid
Pvalb plasmid
Marker
mock
mock
250KDa-
150KDa-
100KDa-
75KDa-
50KDa-
37KDa-
25KDa-
20KDa-
15KDa-
10KDa-
IB: anti-Pvalb
 (12KDa)
Cell
Medium
Supplemental Figure S3. Pvalb expressed in C2C12 muscle cells as a result of plasmid transfection. A Pvalb expression plasmid (OriGene Technologies, Rockville, MD, USA) was transfected into the C2C12 mouse skeletal muscle cell line using the Lipofectamine 3000 transfection reagent (Thermo Fisher Scientific, Waltham, MA, USA) in Dulbecco's Modified Eagle Medium (DMEM) containing 10% fetal bovine serum (FBS). After 24 h, the culture medium was replaced with DMEM containing 1% FBS and incubated for 24 h; the cells and culture medium were collected separately. Cell extracts and culture medium derived from the same number of cells were used for immunoblotting analysis with anti-Pvalb antibodies (Proteintech, Rosemont, IL, USA) after electrophoresis.

## Slide 5
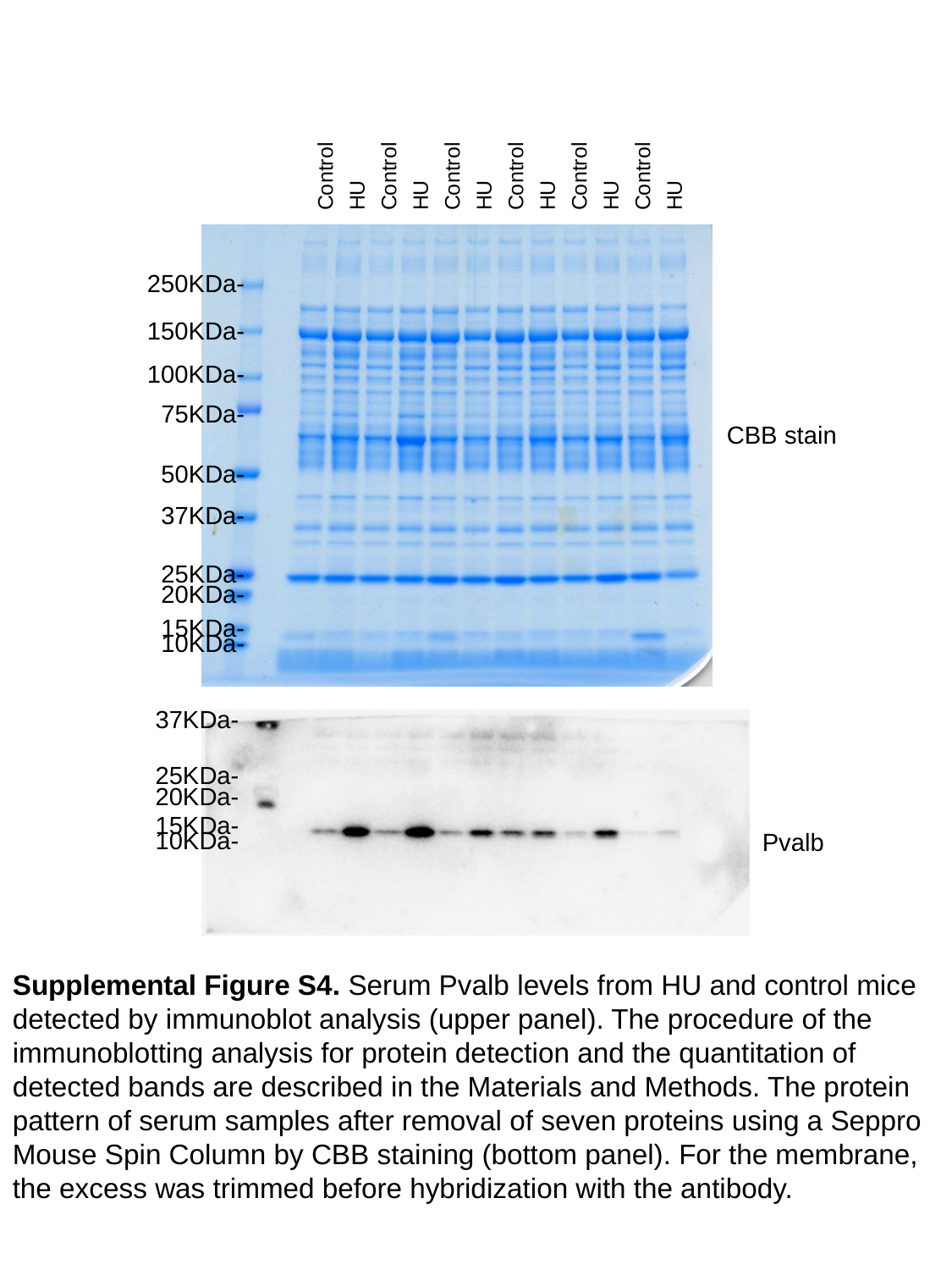

Control
HU
Control
HU
Control
HU
Control
HU
Control
HU
Control
HU
250KDa-
150KDa-
100KDa-
75KDa-
CBB stain
50KDa-
37KDa-
25KDa-
20KDa-
15KDa-
10KDa-
37KDa-
25KDa-
20KDa-
15KDa-
10KDa-
Pvalb
Supplemental Figure S4. Serum Pvalb levels from HU and control mice detected by immunoblot analysis (upper panel). The procedure of the immunoblotting analysis for protein detection and the quantitation of detected bands are described in the Materials and Methods. The protein pattern of serum samples after removal of seven proteins using a Seppro Mouse Spin Column by CBB staining (bottom panel). For the membrane, the excess was trimmed before hybridization with the antibody.

## Slide 6
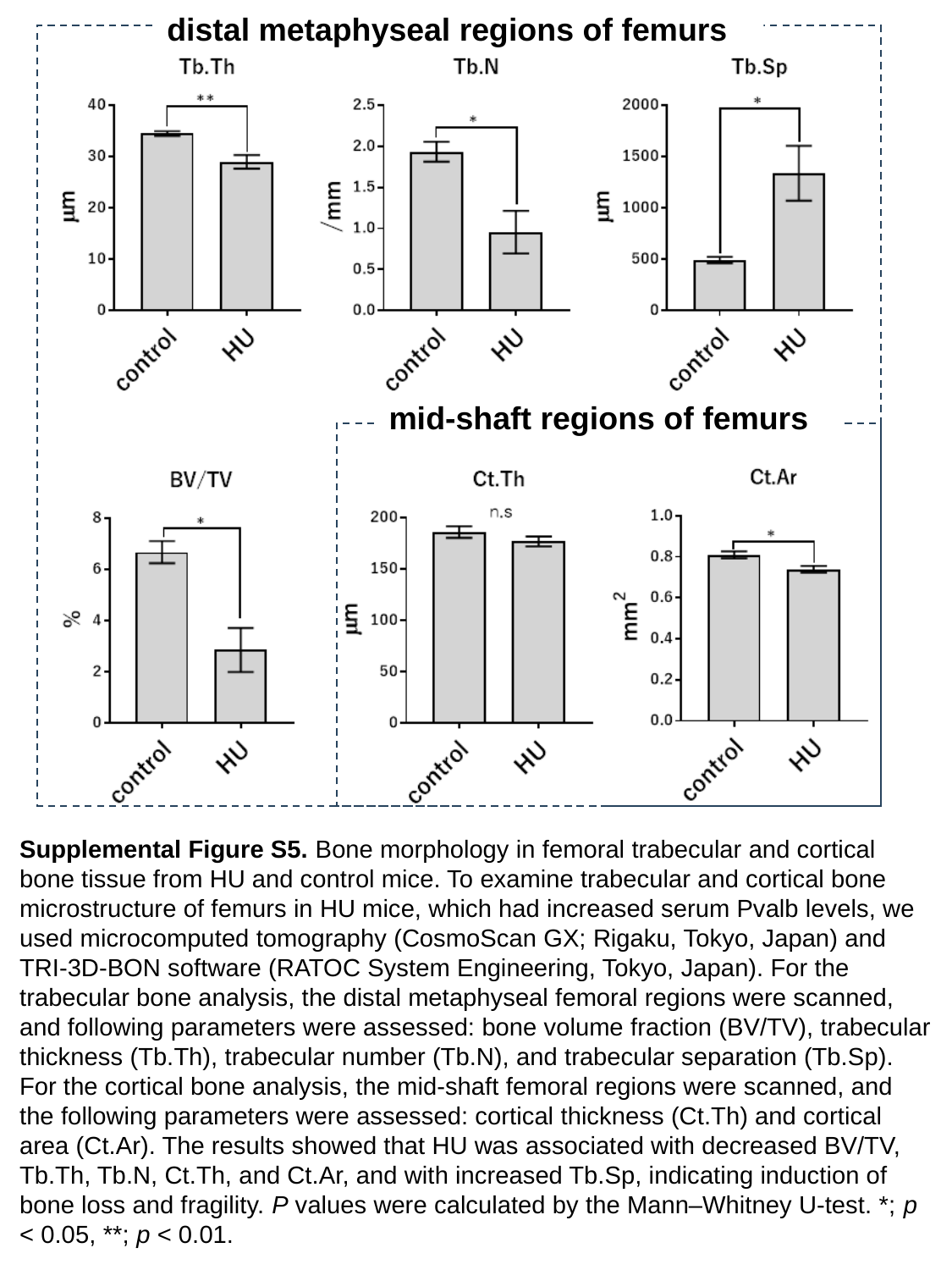

distal metaphyseal regions of femurs
mid-shaft regions of femurs
Supplemental Figure S5. Bone morphology in femoral trabecular and cortical bone tissue from HU and control mice. To examine trabecular and cortical bone microstructure of femurs in HU mice, which had increased serum Pvalb levels, we used microcomputed tomography (CosmoScan GX; Rigaku, Tokyo, Japan) and TRI-3D-BON software (RATOC System Engineering, Tokyo, Japan). For the trabecular bone analysis, the distal metaphyseal femoral regions were scanned, and following parameters were assessed: bone volume fraction (BV/TV), trabecular thickness (Tb.Th), trabecular number (Tb.N), and trabecular separation (Tb.Sp). For the cortical bone analysis, the mid-shaft femoral regions were scanned, and the following parameters were assessed: cortical thickness (Ct.Th) and cortical area (Ct.Ar). The results showed that HU was associated with decreased BV/TV, Tb.Th, Tb.N, Ct.Th, and Ct.Ar, and with increased Tb.Sp, indicating induction of bone loss and fragility. P values were calculated by the Mann–Whitney U-test. *; p < 0.05, **; p < 0.01.
